# Beyond the left cerebral hemisphere: bilateral language lateralization in healthy aging and its clinical implications

**DOI:** 10.3389/fnagi.2025.1547162

**Published:** 2025-07-23

**Authors:** David Toloza-Ramirez, Rodrigo Santibañez, Leonardo Arraño-Carrasco, Romina Zunino-Pesce, Teresa Julio-Ramos, David A. Copland, Camilo Quezada, Carolina Mendez-Orellana

**Affiliations:** ^1^Interdisciplinary Center for Neuroscience, Faculty of Medicine, Pontificia Universidad Católica de Chile, Santiago, Chile; ^2^Faculty of Rehabilitation Sciences, School of Speech Therapy, Exercise and Rehabilitation Sciences Institute, Universidad Andres Bello, Santiago, Chile; ^3^Department of Neurology, Faculty of Medicine, Pontificia Universidad Católica de Chile, Santiago, Chile; ^4^Department of Radiology, Faculty of Medicine, Pontificia Universidad Católica de Chile, Santiago, Chile; ^5^PhD Program in Health Sciences and Engineering, Universidad de Valparaiso, Valparaiso, Chile; ^6^Queensland Aphasia Research Centre, School of Health and Rehabilitation Sciences, University of Queensland, Brisbane, QLD, Australia; ^7^Departamento de Fonoaudiología, Facultad de Medicina, Universidad de Chile, Santiago, Chile; ^8^Faculty of Medicine, Health Sciences School, Pontificia Universidad Católica de Chile, Santiago, Chile; ^9^Department of Neurosurgery, Faculty of Medicine, Pontificia Universidad Católica de Chile, Santiago, Chile; ^10^Department of Radiology and Nuclear Medicine, Erasmus MC – University Medical Center Rotterdam, Rotterdam, Netherlands

**Keywords:** aging, semantics, phonology, neural basis, functional MRI

## Abstract

**Background:**

Functional MRI (fMRI) studies conducted on young adults reveal a predominantly left-lateralized cortical language network during semantic and phonological processing (SP and PP, respectively). Both linguistic dimensions have been advanced as potential cognitive markers of pathological aging. However, the neural mechanisms underlying SP and PP among healthy older adults remain poorly understood.

**Aim:**

This study aimed to investigate the dynamics of language lateralization among native Spanish-speaking older adults in relation to their behavioral performance in specific semantic and phonological tasks.

**Methodology:**

Twenty-eight healthy, right-handed older Chilean adults (mean age: 67.7, SD±: 7.44, range: 60–87) took part in an fMRI session during which they performed semantic and phonological tasks. They were also evaluated for overall language performance using the Spanish version of ScreeLing and verbal fluency tasks. A fixed-effect analysis was performed to explore group-level differences. Standard regression analyses were also used to assess the association between brain activation and language performance.

**Results:**

Both SP and PP elicited bilateral activation in the pars triangularis and opercularis of the inferior frontal gyrus (IFG) and the superior temporal gyrus. Activation was also observed in the left inferior parietal gyrus. Semantic fluency performance was significantly associated with activation in the right angular gyrus and the pars opercularis of the IFG. In contrast, phonological fluency was associated with bilateral activation in the IFG pars orbitalis.

**Conclusion:**

Among healthy older adults, SP and PP recruit bilateral language-related brain regions, potentially reflecting compensatory mechanisms associated with normal aging. Notably, the IFG pars orbitalis may play a distinct role in supporting phonological fluency, despite not being a region traditionally linked to PP. Further research is needed to clarify the contribution of this region to phonological performance among aging adults.

## Introduction

Aging is commonly linked to declines in memory, attention, executive functions, and processing speed (Spaan and Dolan, 2010; Harada et al., 2013). While all of these domains are typically of high interest in studies addressing cognitive aging, language skills are somewhat overlooked, despite also undergoing meaningful changes (Hartsuiker and Barkhuysen, 2006). Increased hesitations, word-finding difficulties, and disfluencies are frequently reported in spontaneous speech among healthy older adults and often reflect underlying deficits in semantic and phonological processing (SP and PP, respectively) (Gordon and Kindred, 2011; Hartsuiker and Barkhuysen, 2006; Kavé and Knafo-Noam, 2015).

Clinical practice often largely focuses on semantic deterioration when addressing dementia. However, emerging evidence suggests that PP may also play a crucial role in dementias and should receive more attention (Olmos-Villaseñor et al., 2023). SP and PP are critical components of language that have received growing attention in aging and clinical neuroscience (Peelle et al., 2014). These processes are not only relevant for understanding the trajectory of normal aging but also serve as key markers for early detection and differentiation of neurodegenerative conditions, including several forms of dementia (Jiskoot et al., 2023). In fact, alterations in SP and PP are among the earliest cognitive signs observed in pathological aging and are often used to differentiate between subtypes of Primary Progressive Aphasia (PPA) and other language-variant dementias (Toloza-Ramírez et al., 2021).

Functional MRI (fMRI) is a key neuroimaging technique that allows examining the neural substrates of language processing in both healthy individuals and clinical populations. Many fMRI studies (conducted mainly with healthy young participants) have explored the activation patterns that emerge during SP and PP. Results show SP to be associated with a bilateral network involving both hemispheres, particularly the temporal lobes, while PP has been consistently linked to left-lateralized fronto-parietal circuits (Heim et al., 2008, 2009; Hickok and Poeppel, 2007; Kielar et al., 2011; Meinzer et al., 2009; Wagner et al., 2014). Specifically, the inferior frontal gyrus (IFG), superior temporal gyrus (STG), and middle temporal gyrus (MTG) have been reported to be activated by both SP and PP tasks (McDermott et al., 2003; Sekiyama et al., 2003; Rimol et al., 2005; Abel et al., 2009; de Zubicaray and McMahon, 2009). Differential activation patterns have also been observed. Broca’s area appears to be more engaged during PP (Wagner et al., 2014), whereas regions such as the posterior left middle frontal gyrus and the precuneus have been more selectively associated with SP (Meinzer et al., 2009; Zhuang et al., 2016).

Compared to pathological aging, the unimpaired neural basis of SP and PP in older adults remains less well understood (Garcia et al., 2022). Some neuroimaging studies have stressed the need to further examine how SP and PP evolve with age and what these changes reveal about compensatory versus neurodegenerative mechanisms (Shafto and Tyler, 2014; Joyal et al., 2020). Age-related changes in language networks, such as reduced lateralization or increased recruitment of contralateral regions, have been interpreted as compensatory adaptations (Crosson et al., 2007; Kielar et al., 2011). These adaptations may initially support preserved language function but can also signal early neural inefficiencies that precede clinical symptoms. Similar activation patterns have been reported for stroke (Saur et al., 2006) and brain tumor lesions (Duffau, 2020). Increased recruitment of right-hemisphere language homologs is often interpreted as maladaptive when it is associated with poor language outcomes or disorganized network reconfiguration (Heiss et al., 1999; Saur et al., 2006). For instance, Heiss et al. (1999) found that persistent right-hemisphere activation in post-stroke aphasia patients was linked to less efficient language recovery, suggesting maladaptive rather than compensatory reorganization. Likewise, Saur et al. (2006) reported that right-hemisphere recruitment was prominent during the subacute phase post-stroke but decreased during recovery, reinforcing the view that excessive or persistent right-hemisphere activation may be maladaptive. However, among healthy older adults, right-hemisphere activation is not necessarily maladaptive or pathological. On the contrary, it might reflect typical neuroplastic adaptations that support language processing in the face of structural and functional age-related decline (Cabeza et al., 2002; Berlingeri et al., 2013; Gajardo-Vidal et al., 2018). Recent neuroimaging studies have reported positive associations between language performance and structural or functional integrity in right-hemisphere regions, including inferior frontal and superior temporal gyrus (Solomons et al., 2025; Gajardo-Vidal et al., 2018). These findings suggest that right-hemisphere engagement in healthy aging may play a beneficial role in maintaining language skills (Cabeza et al., 2002). Furthermore, age-related changes in activation patterns during SP and PP have been widely reported, suggesting altered network efficiency and reduced hemispheric lateralization (Diaz et al., 2014). Yet, the functional interpretation of such bilateral activation remains open to debate. While some authors argue that bilateral recruitment reflects increased cognitive effort or decreased neural specificity (Reuter-Lorenz and Cappell, 2008), others suggest that it should be viewed as a compensatory mechanism that helps sustain linguistic performance, as proposed by the hemispheric asymmetry reduction in older adults (HAROLD) model (Cabeza et al., 2002; Meunier et al., 2014). Thus, it remains unclear whether the engagement of specific brain regions during SP and PP among healthy aging individuals reflects a compensatory mechanism, an early marker of inefficiency, or a normal variation. Moreover, little is known about whether the magnitude of activation in these regions is linked to behavioral performance during the execution of language tasks, an important consideration if such neural signatures are to be considered early markers of cognitive decline.

The design of fMRI language tasks plays a pivotal role in determining observed activation patterns (Black et al., 2017; Hoffman and Morcom, 2018; Price, 2000). fMRI tasks that require internal speech (covert tasks) are widely used in the neuroimaging of language processing because they minimize head motion (Berro et al., 2021; Huang et al., 2002). However, such tasks may present a major disadvantage: internal speech cannot be monitored. Thus, it is difficult to ascertain whether participants performed the task correctly and whether the resulting language activation is reliable (Thakkar et al., 2022). This is crucial, especially when studying healthy aging adults and clinical populations, such as in the context of language lateralization prior to brain tumor resection. fMRI tasks that include responses via an MRI-compatible device are easier to implement and allow researchers to control task performance, as participants are able to respond by pressing buttons.

Most neuroimaging studies addressing SP and PP focus on relatively young, healthy individuals, which limits the applicability of findings to clinical populations affected by conditions such as dementia. In addition, most of these fMRI studies of language processing have been conducted with English-speaking participants (Zhuang et al., 2016; Rizio et al., 2017; Diaz et al., 2019), which further limits the generalizability of the findings. Although some studies have been conducted with Spanish-speaking participants (e.g., Prado et al., 2023), most have not included participants from Latin American countries, thereby excluding relevant perspectives. Differences in sociocultural background, orthography, or phonology may influence both the cognitive mechanisms and neural networks that support language processing (Kovelman et al., 2008a, 2008b; Costa and Sebastián-Gallés, 2014). Both neuroimaging and behavioral studies focusing on Spanish-speaking individuals from Latin American countries are rare (Toloza-Ramirez et al., 2023).

In the present study, we aim to investigate the dynamics of language lateralization among native Spanish-speaking older adults in relation to their behavioral performance in specific semantic and phonological tasks. We implemented fMRI language tasks that could be monitored and controlled in real time during MRI acquisition. We expect to contribute to the understanding of how these two processes, considered key markers of pathological aging, can be used for early detection and differentiation of neurodegenerative conditions in Spanish-speaking individuals.

Thus, this study addresses two key questions: (1) What are the language-related brain regions recruited during semantic and phonological processing (SP and PP) in healthy older adults using a validated covert auditory fMRI task? (2) Are individual differences in neural activation in these regions associated with differences in behavioral language performance? Based on previous evidence and compensatory models such as HAROLD, we hypothesized that healthy older adults would exhibit reduced language lateralization, with bilateral recruitment during both tasks. Moreover, we expected that greater activation in right-hemisphere language regions would be positively associated with better performance on semantic and phonological tasks.

## Materials and methods

### Participants

Twenty-eight healthy native Spanish-speaking older adults (mean age = 67.7 years, SD = ±7.44, range = 60–87 years) were recruited for the study (see Table 1). Inclusion criteria were the following: (a) age ≥ 60 years, being monolingual (Spanish as a native language), (b) being right-handed, which was assessed with the Edinburgh Handedness Inventory (EHI) (Oldfield, 1971), (c) having a balanced body mass in order to fit into the magnetic resonance (MR) scanner, (d) not having any medical contraindications for MR imaging, (e) scoring ≥ 29 points on the Chilean Spanish version of the abbreviated Token Test (Julio-Ramos et al., 2024a), and (f) scoring ≥ 21 points on the Spanish version of the MoCA Test (Delgado et al., 2019) to control for the possible presence of mild cognitive impairment. Exclusion criteria were: (a) presence of severe hearing or visual perceptual deficits, (b) a clinical history of neurological or psychiatric conditions, including any recent psychiatric illness, and (c) family history of Alzheimer’s disease or other types of dementia. This research project was approved by the Medical Ethics Committee of the Pontificia Universidad Católica de Chile (ID 220322003). All participants signed an informed consent prior to participation.

**Table 1:** Participant information.

	Sample
	(*n* = 28)
Age	
years, mean (SD)	67.7 (±7.44)
[range]	[60–87]
Education	
years, mean (SD)	14.0 (±3.19)
[range]	[7–19]
Sex, *n (%)*	
Female	16 (57.1)
Male	12 (42.9)
Level of education, *n (%)*	
≤12 years	10 (35.7)
>12 years	18 (64.3)
ScreeLing, mean (SD)	
Semantic	23.7 (±0.55)
Phonology	23.6 (±0.56)
Total	70.3 (±1.64)
Phonological fluency, mean (SD)	
FAS-F	14.2 (±4.13)
FAS-A	13.3 (±5.56)
FAS-S	15.2 (±5.5)
FAS-Total	42.5 (±13.69)
Semantic fluency, mean (SD)	
Animals	20.4 (±4.48)
Professions	12.8 (±4.11)
Total	33.2 (±7.61)
In- scanner fMRI task performance, % correct responses	
Semantic association task	98%
Rhyming decision task	96%

SD, Standard Deviation.

### Language assessments

To interpret functional brain activation and explore brain–behavior correlations, all participants completed a language test battery specifically designed for SP and PP. We selected psychometrically validated language scales suited for Spanish-speaking individuals.

***Verbal fluency tasks:*** Phonological and semantic verbal fluency were assessed using standardized norms for Spanish-speaking adults (Olabarrieta-Landa et al., 2015), considering an average of 15 words per minute. Participants were asked to produce as many words as possible within 60 s under two conditions: (a) phonological fluency, using the initial letters F, A, and S; and (b) semantic fluency, with categories such as animals and professions.

***ScreeLing test:*** The ScreeLing is a screening instrument designed to assess language deficits in clinical subtypes of frontotemporal dementia and Alzheimer’s dementia, assessing Syntax, Phonology, and Semantics. We used the validated version for Chilean Spanish speakers (Mora-Castelletto et al., 2020). Each linguistic level may provide a maximum of 24 points, with a total score of 72. A cut-off score of 68 signals potential impairment, with lower scores suggesting compromised language function.

### Magnetic resonance imaging

All scanning was performed at the Radiology Department of Hospital Clínico Pontificia Universidad Católica de Chile. Volunteers underwent both functional MRI (fMRI) and conventional structural MRI.

***Data acquisition:*** Phillips Ingenia 3.0 T MRI system was used. An axial high-resolution 3D inversion recovery fast-spoiled gradient echo T1-weighted sequence was acquired with the following parameters: echo time (TE) = 2.1 ms; repetition time (TR) = 6.1 ms; inversion time (TI) = 450 ms; flip angle = 12°; matrix = 256 × 224; field of view (FOV) = 24 × 18 cm; slice thickness = 0.8 mm; no interslice gap. Functional scans were acquired using a gradient-echo planar imaging pulse sequence as follows: TE = 30 ms; TR = 3,000 ms; flip angle = 90°; matrix = 96 × 96; FOV = 24–26 cm; slice thickness = 3.0 mm; no interslice gap.

***fMRI language paradigms:*** fMRI tasks were adapted for Spanish-speaking individuals and specifically designed to assess SP and PP (Thakkar et al., 2022; Méndez-Orellana et al., 2021). To assess SP, we employed a semantic association task (see Supplementary Figure 1A). Each experimental block presented six noun pairs, three of them semantically related and the remaining three semantically unrelated, displayed in random sequence. In the control condition, participants heard six auditory stimuli—three pure tones (500 Hz) and three Brownian noise bursts—also randomized. Participants were instructed to press a response button when they heard a semantically related word pair and to withhold responses for unrelated pairs. During the control condition, the button press was triggered only upon hearing Brownian noise. PP was evaluated using a rhyme judgment task (Supplementary Figure 1B), which followed the same block structure as the semantic association task but used rhyming and non-rhyming word pairs instead. Each task block lasted 21 s and was repeated six times, alternating with its respective control block. All word stimuli across both tasks were thoroughly controlled for lexical frequency, word length, and imageability to ensure experimental consistency. The task timing parameters, including the repetition time (TR), were determined based on performance data from a previous study involving 80 healthy older adults (Méndez-Orellana et al., 2021), ensuring the protocol was optimized for the targeted population. Stimuli presentation and response recording were managed using Presentation software (Neurobehavioral Systems). Prior to scanning sessions, participants completed a training phase using a laptop and a separate set of word pairs to familiarize themselves with the tasks. The percentage of correct responses during scanning is shown in Table 1.

***Image processing:*** All raw DICOM images were converted to NIfTI format using MRIcron software (Penny et al., 2011). Task-based fMRI data were analyzed using SPM12 (Wellcome Trust Centre for Neuroimaging, London, UK) (Friston, 2007) in MATLAB R2023a software (MathWorks, Inc.). Experimental data from all volunteers were spatially pre-processed. Preprocessing steps included: (a) manual realignment for motion correction, (b) coregistration of the functional scan to each participant’s high-resolution T1-weighted structural image (Friston et al., 1995), (c) segmentation of T1 images and spatial normalization of both functional and anatomical scans to Montreal Neurological Institute (MNI) template space using the unified segmentation approach (Ashburner and Friston, 2005; Crinion et al., 2007), (d) normalized functional images were resampled to 3 × 3 × 3 mm^3^; anatomical images were resampled to 1 × 1 × 1 mm^3^, and (e) spatial smoothing was applied to functional images using 6 × 6 × 6 mm^3^ full-width at half maximum (FWHM) 3D Gaussian kernel to improve signal-to-noise ratio and reduce anatomical variability (Friston et al., 1999). Statistical activation maps were generated using a general linear model (GLM) to assess task-related neural responses.

### fMRI data analysis

***MNI coordinates extraction:*** To quantify peak activations in language-relevant brain regions, we used the MarsBaR toolbox in SPM12 (Brett et al., 2002). Regions of interest (ROI) were defined based on second-level activation maps from the auditory semantic decision and rhyming decision tasks, which targeted SP and PP, respectively. MNI coordinates corresponding to peak activation were extracted to generate spherical ROIs for each fMRI task separately. Each sphere had a diameter of 5 mm, centered at the identified peak coordinate. For each ROI, we extracted mean beta values from the contrast images generated during second-level analysis, using a global scaling factor with a grand mean of zero. In this study, brain activation refers to beta values extracted from ROIs defined at the second-level group analysis, which were applied to contrast images at the individual level to examine condition-specific. We focused on eight core language-related ROIs: IFG (pars triangularis, orbitalis, and opercularis), supplementary motor area (SMA), superior and middle temporal gyrus (STG and MTG, respectively), angular gyrus, and supramarginal gyrus. ROI masks were created bilaterally using the WFU PickAtlas toolbox, resulting in a total of 16 masks (left and right hemispheres). For all fMRI analyses, a significance threshold of *α* = 0.05 was adopted.

### Statistical analysis

All statistical analyses were conducted using R software (R Core Team, 2024). Descriptive statistics were calculated to summarize demographic characteristics and performance for language assessments. Continuous variables (e.g., age, test scores) are reported as means with standard deviations, whereas categorical variables (e.g., sex) are presented as counts and corresponding percentages.

Given the modest sample size, which limited the statistical power to fit fully adjusted models including all relevant covariates (i.e., sex, age, and years of education), we employed a sequential regression approach to examine the relationship between language performance and neural activation while minimizing the risk of model overfitting.

In the first step, and consistent with the primary aim of the study, we conducted a series of univariate linear regression analyses, in which each behavioral measure (for semantic and phonological tasks) was used as a single predictor and regressed independently against the mean beta values extracted from all predefined ROIs. These ROIs were defined based on peak activations obtained from second-level fMRI analyses of the semantic and phonological tasks. We focused on 16 ROIs in total: the IFG (pars triangularis, opercularis, and orbitalis), SMA, MTG, STG, angular gyrus, and supramarginal gyrus, all considered bilaterally. Spherical ROI masks (5 mm diameter) were created using MNI coordinates and applied using the WFU PickAtlas and MarsBaR toolboxes. This procedure served as an omnibus screening method to identify brain regions whose activation was significantly associated (*p* < 0.05) with any of the observed behavioral measures. To maintain statistical rigor and avoid double-dipping bias, we included demographic variables (age, sex, and years of education) in the regression models when they showed statistically significant effects in the relevant analyses.

In the second step, having identified relevant ROIs, we implemented additional simple linear regressions to evaluate whether demographic variables (namely sex, age, and years of education) individually predicted activation in the previously identified ROIs. This step was crucial to determine whether any of these demographic variables needed to be controlled for. None of the demographic variables significantly predicted activation in any ROI individually. To further control for possible interaction among demographic variables, we regressed a three-predictor model including sex, age, and years of education, against values of each identified ROI. Only one demographic variable was found to significantly impact activation values in this step, namely, years of education for the right IFG pars orbitalis. Consequently, this particular variable was controlled for in all subsequent two-predictor models implemented when examining the influence of all behavioral measures. Reported linear models were inspected to verify standard assumptions, including normality of residuals, linearity, homoscedasticity, and absence of multicollinearity. Variance inflation factor (VIF) values were examined to rule out redundancy among predictors. To enhance robustness and account for sampling variability, bias-corrected and accelerated (BCa) bootstrap confidence intervals were calculated for all statistically significant models, using 10,000 iterations. Final model estimates, including *f*^2^ effect size (Cohen, 1988), standard errors, *t* and *p* values, and confidence intervals (95% level) are reported in Supplementary Table 4. ROI-based analyses were the primary focus of the study; however, exploratory whole-brain analyses were also conducted to complement ROI findings.

## Results

### Neural activation underlying SP and PP

Twenty-six healthy older adults were included in the final analysis. Two participants were excluded due to excessive head motion that did not meet realignment criteria. To characterize brain activation associated with SP and PP, separate fixed-effects analyses were performed in SPM12 for each fMRI task. We report statistically significant clusters at *p* < 0.05, FWE-corrected at the cluster level (*k* = 10). Activation patterns are summarized in Table 2 and 3.

**Table 2:** Activation pattern for SP in healthy older adults.

Anatomical location	Side	BA	Cluster size	Peak MNI			*T*-value	*p* value
				*x*	*y*	*z*		
Cerebellum	R		391	30	−61	−29	10.75	0.000
Middle temporal gyrus	R	21	101	60	−25	−2	9.85	0.000
Middle temporal gyrus	L	22	157	−60	−19	−5	9.47	0.000
Inferior temporal gyrus	L	37	30	−51	−55	−14	9.19	0.000
Insula	L	47	45	−27	26	−5	8.69	0.000
Inferior frontal gyrus (triangularis)	L	45	23	−51	29	19	8.02	0.000
Supplementary motor area	R	8	21	6	20	49	7.55	0.001
Precentral gyrus	L	44	30	−45	8	31	7.55	0.001
Superior temporal gyrus	R	22	3	54	−1	−8	7.20	0.002
Lingual	L	18	14	−9	−49	1	7.12	0.002
Calcarine	L	18	11	−18	−73	7	7.08	0.003
Middle cingulum	R	32	9	9	26	37	7.05	0.003
Middle frontal gyrus	L	44	6	−51	14	40	6.91	0.004
Inferior frontal gyrus (opercularis)	L	48	1	−51	17	16	6.67	0.007
Cerebellum	L		10	−9	−76	−32	6.50	0.011
Middle occipital gyrus	L	7	6	−27	−64	37	6.45	0.013
Inferior parietal gyrus	L	7	1	−30	−73	46	6.44	0.013
Cerebellum	L		4	−39	−70	−29	6.42	0.014
Postcentral gyrus	L	4	1	−57	−7	40	6.40	0.015
Superior temporal gyrus	R	48	1	57	−4	−2	6.24	0.022
Medial superior frontal gyrus	L	32	3	−3	26	43	6.12	0.030
Cerebellum	L	19	2	−24	−61	−26	6.01	0.040
Cerebellum	L	18	2	−9	−76	−20	5.96	0.045

L, left hemisphere; R, Right hemisphere; BA, Brodmann area; MNI, Montreal Neurological Institute coordinates. Results reported at *p* < 0.05, FWE-corrected at the cluster level (*k* = 10).

**Table 3:** Activation pattern for PP in healthy older adults.

Anatomical location	Side	BA	Cluster size	Peak MNI			*T*-value	*p*-value
				*x*	*y*	*z*		
Superior temporal gyrus	R	22	216	57	−1	−8	10.83	0.000
Superior temporal gyrus	L	22	112	−60	−22	4	9.51	0.000
Cerebellum	R		218	18	−73	−41	9.09	0.000
Inferior frontal gyrus (triangularis)	L	48	149	−42	14	25	8.11	0.000
Middle temporal gyrus	L	22	27	−54	−40	4	7.96	0.000
Inferior temporal gyrus	L	37	8	−51	−55	−14	7.9	0.000
Supplementary motor area	R	8	45	3	23	52	7.53	0.001
Inferior frontal gyrus (triangularis)	R	48	23	45	29	25	7.34	0.001
Insula	L	47	13	−30	20	−2	6.83	0.005
Precentral gyrus	L	6	8	−45	5	49	6.8	0.005
Vermis	R		4	0	−58	−20	6.74	0.006
Inferior frontal gyrus (opercularis)	R	44	10	45	11	31	6.74	0.006
Inferior parietal gyrus	L		1	−39	−58	58	6.44	0.013
Precentral gyrus	L	6	1	−45	−4	52	6.35	0.017
Inferior frontal gyrus (opercularis)	L	48	3	−51	14	7	6.18	0.026
Middle frontal gyrus	L	9	1	−48	14	43	6.17	0.027
Anterior cingulum	L		1	−9	23	28	6.15	0.029
Cerebellum	L		6	−12	−76	−26	6.14	0.029
Superior parietal gyrus	L	7	2	−33	−64	58	6.12	0.031
Cerebellum	L		2	−12	−64	−26	6.1	0.032
Insula	R	47	2	33	20	−5	6.07	0.034

L, left hemisphere; R, Right hemisphere; BA, Brodmann area; MNI, Montreal Neurological Institute coordinates. Results reported at *p* < 0.05, FWE-corrected at the cluster level (*k* = 10).

For the semantic decision task (see Table 2), the analysis revealed a bilateral activation pattern. Core language regions involved included left IFG (pars triangularis and opercularis), right SMA, bilateral MTG, right STG, and left inferior parietal gyrus. Additional activation was observed in the insula, precentral and postcentral gyrus, cerebellum, lingual, and middle frontal gyrus, suggesting a widespread engagement of fronto-temporo-parietal and subcortical regions during SP.

In contrast, the rhyming judgment task (see Table 3) also elicited a bilateral activation pattern for older adults, but with partially distinct topography. Significant clusters were found in bilateral IFG (pars triangularis and opercularis), bilateral STG, left MTG, and the left inferior and superior parietal gyrus. Notably, the right SMA, insula, precentral gyrus, anterior cingulate cortex, and cerebellum were also recruited. Compared to SP, PP showed more prominent engagement of right-hemisphere temporal and frontal regions, particularly the right IFG, and increased involvement of parietal cortices, suggesting a differential lateralization and regional specificity between SP and PP.

Before exploring neural-behavioral associations, we examined participants’ behavioral performance on standardized language tasks. As summarized in Table 1, participants showed high scores across all subdomains of the ScreeLing. Semantic (mean = 23.7, SD = ±0.55) and phonological (mean = 23.6, SD = ±0.56) domains approached the expected scores for healthy adults, and total scores were uniformly high (mean = 70.3, SD = ±1.64), suggesting preserved core linguistic skills. On verbal fluency, phonological fluency scores (FAS total: mean = 42.5, SD = ±13.69) fell within the expected range for Spanish-speaking older adults, with performance relatively consistent across the three letters (*F* = 14.2, *A* = 13.3, *S* = 15.2). In contrast, semantic fluency scores (Total: mean = 33.2, SD = ±7.61) showed a slightly different overall performance, with the animals category (mean = 20.4, SD = ±4.48) better than the professions category (mean = 12.8, SD = ±4.11).

### Neural and language performance

**Figure 1:**
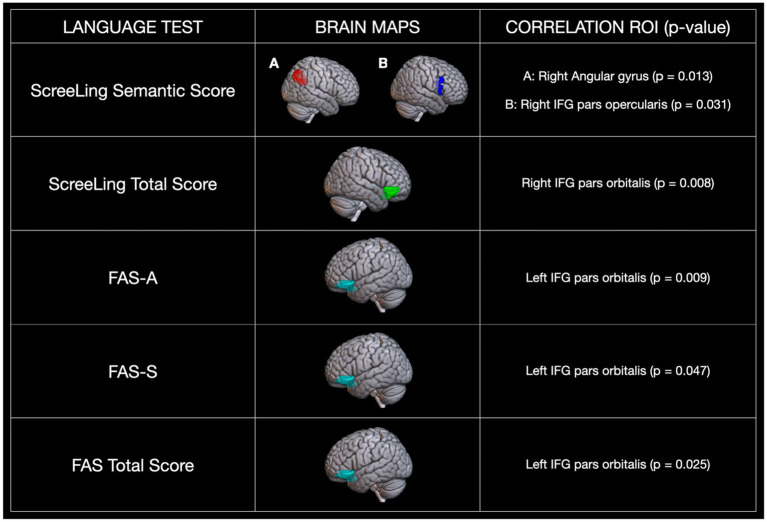
Regression analysis for neural activation and behavioral performance. Significance: *p* < 0.05.

To examine the relationship between neural activation and language performance, we conducted a series of single-predictor linear regression models using beta values extracted from functionally defined ROIs. Figure 1 and Supplementary Table 4 summarize all significant associations.

For the semantic decision task, activation in the right angular gyrus (*p* = 0.013) and the right IFG pars opercularis (*p* = 0.031) was significantly predicted by the ScreeLing semantics subdomain score. For the auditory rhyming decision task (phonological task), activation in the right IFG pars orbitalis was significantly predicted by ScreeLing total score (*p* = 0.008), while activation in the left IFG pars orbitalis was predicted by performance on phonological fluency [FAS-A (*p* = 0.009), FAS-S (*p* = 0.047), and total score (*p* = 0.025)]. These findings show that different language-related ROIs are selectively sensitive to semantic or phonological performance and reflect lateralized contributions from both hemispheres.

## Discussion

We aimed to investigate the dynamics of language lateralization among native Spanish-speaking older adults in relation to their behavioral performance in specific semantic and phonological tasks. Our findings revealed that both SP and PP recruit a bilateral network of frontal, temporal, and parietal regions, including the IFG, SMA, MTG, STG, and inferior parietal gyrus. Importantly, PP elicited additional activation in the superior parietal region. Overall, a pattern of bilateral activation was observed for both phonological and semantic processing. Moreover, the association between neural activation and behavioral performance (across both observed tasks) was significant in specific regions, namely the bilateral pars opercularis of the IFG, right angular gyrus, and bilateral IFG (pars orbitalis), suggesting that these areas may play a key role in supporting language function in healthy aging.

### Semantic and phonological processing among older adults

It has been claimed that bilateral activation of the IFG, particularly the pars triangularis and opercularis, aligns with their established roles in lexical access and semantic selection (Binder et al., 2009). Likewise, the involvement of bilateral MTG and right STG supports their contributions to semantic integration, lexical retrieval, auditory perception, and the processing of complex linguistic information (Petersen et al., 1990; Papeo et al., 2019). Moreover, activation in the inferior parietal gyrus and the cerebellum may reflect the integration of semantic and motor representations, as suggested by Stoodley and Schmahmann (2010).

Previous studies have reported variability in the lateralization patterns associated with SP. Carpentier et al. (2001) observed bilateral activation in the IFG (pars triangularis and opercularis) and STG, whereas Zahn et al. (2000) reported left-lateralized activation in the IFG pars triangularis, MTG, and angular gyrus, along with additional right precentral gyrus activation among older adults. Comparative studies show that younger adults exhibit predominantly left-lateralized activation, while older adults show more bilateral recruitment, potentially reflecting compensatory mechanisms, as suggested by Davis et al. (2008). These authors underscore increased frontal and parietal activity, such as left IFG pars triangularis and right supramarginal gyrus, among older adults, which may be associated with reduced activation in posterior brain areas.

The observed bilateral activation may be interpreted through the lens of the HAROLD model, which posits that aging is associated with reduced hemispheric asymmetry, leading to greater bilateral recruitment to maintain cognitive performance (Cabeza et al., 2002). This compensatory activation may counteract age-related declines in neural efficiency, consistent with some studies (Peelle et al., 2010; Tyler et al., 2010) showing that younger adults typically exhibit greater left-lateralized activation in regions such as the IFG and MTG, reflecting more specialized and efficient semantic networks.

In contrast to SP, PP exhibited additional activation in the superior parietal region, suggesting an increased reliance on attentional and working memory resources. While young adults typically show left-lateralized activation in the STG and inferior parietal gyrus during PP tasks (Liebenthal et al., 2013; Liu et al., 2022), our findings show a pattern of bilateral activation among older adults, involving IFG, STG, and SMA. This pattern is consistent with previous studies (Liu et al., 2009; Steinbrink et al., 2012; Lim et al., 2014; Jung et al., 2017).

The increased bilateral activation observed among older adults may reflect a compensatory recruitment of additional neural resources to mitigate age-related decreases in PP efficiency (Aerts et al., 2013; Diaz et al., 2014; Meunier et al., 2014). In fact, PP has been shown to be particularly vulnerable to aging, with studies reporting alterations in the BOLD signal and greater recruitment of additional brain regions (Cabeza and Dennis, 2013; Eckert et al., 2010; Peelle et al., 2010). Froehlich et al. (2018) reported increased activation in the angular and supramarginal gyrus in older adults, although our study did not observe activation in these areas. Such discrepancies may be the result of differences in tasks or sample traits.

These compensatory activations may be necessary to offset reduced efficiency in the core phonological network and are consistent with the bilateral activation pattern observed in our study. One possible explanation for this phenomenon is provided by the transmission deficit hypothesis, which suggests that aging weakens the connections between semantic and phonological representations, prompting the brain to rely more heavily on alternative neural pathways and compensatory mechanisms (Park and Reuter-Lorenz, 2009). Additionally, PP may place greater demands on motor control and articulatory planning in the case of older adults, potentially explaining additional activation of regions not typically associated with PP, such as the cerebellum, insula, and precentral gyrus. This interpretation is consistent with the work by Marvel and Desmond (2010), who stress the cerebellum’s role in both cognitive and motor components of language processing, particularly under conditions of increased task complexity or compensatory recruitment.

Importantly, our findings align with the compensation-related utilization of neural circuits hypothesis, which posits that older adults recruit additional neural resources to maintain performance levels as task-related demands increase (Reuter-Lorenz and Cappell, 2008). This view complements the HAROLD model and further underscores the idea of compensatory recruitment. Moreover, behavioral studies have consistently shown that older adults adapt their language processing strategies to bolster performance by leveraging, for instance, contextual and semantic cues (Wingfield and Grossman, 2006; van Boxtel and Lawyer, 2021). Manenti et al. (2013) also showed that higher-performing older adults display more balanced prefrontal activation, which aligns with the idea of flexible and efficient compensatory mechanisms. These behavioral compensations integrate seamlessly with the neural patterns we observed, reinforcing the view that successful language processing during aging involves a dynamic interplay of both neural and behavioral adaptations.

### Brain regions underlying behavioral performance

The angular gyrus has been consistently implicated in SP, contributing to conceptual integration and semantic retrieval (Binder et al., 2009; Seghier, 2013). While traditionally associated with left-lateralized activation, particularly during sentence-level and syntactical processing (Petersson et al., 2012; Zhu et al., 2012), a growing body of evidence strongly suggests a more nuanced role for the right angular gyrus. Seghier (2013) notes that the right angular gyrus is recruited during complex semantic tasks that require the integration of multiple sources of information. Such lateralization may be shaped by task demands and cognitive load, as supported by fMRI studies and meta-analyses indicating predominantly left-sided activation during semantic tasks while acknowledging right-hemisphere involvement under increased cognitive demands (Price et al., 2016; Kuhnke et al., 2020; Jackson, 2021). Our findings align with Binder et al. (2009), who reported right angular gyrus activation during SP, and are further supported by neuroimaging evidence establishing selective difficulty effects on this region during semantic fluency tasks in healthy older adults (Martin et al., 2021; Kuhnke et al., 2023).

Within the context of aging, functional neuroimaging studies have revealed increased right-hemisphere activation, particularly in the IFG, during semantic tasks. Wierenga et al. (2008) and Shafto et al. (2010) observed more extensive right IFG activation among older adults as compared to younger individuals, which correlated with performance on semantic tasks. However, this relationship is not unequivocal; Meinzer et al. (2009) reported that higher semantic fluency performance among older adults does not necessarily predict greater right IFG activation. Instead, they found that lower performance was associated with heightened activity in the right middle frontal gyrus, suggesting that right-hemisphere recruitment may reflect compensatory mechanisms responding to diminished efficiency of canonical language networks.

As for PP, our study identified negative correlations between behavioral performance and activation in the bilateral IFG pars orbitalis during phonological decision tasks. Geva et al. (2012) reported increased activation in the right IFG pars orbitalis and triangularis among older adults. However, only the right pars triangularis was positively correlated with phonological task error rates. Conversely, Gauthier et al. (2009) found that reduced activation in the right IFG was associated with better performance on phonological fluency tasks, supporting the idea that efficient PP depends less on right-hemisphere engagement. This interpretation is further supported by clinical studies involving individuals with left-hemisphere damage, which have shown that increased right IFG activity correlates with poorer phonological fluency, indicating possible maladaptive compensation (Winhuisen et al., 2005, 2007; Saur et al., 2006; Raboyeau et al., 2008).

Traditionally, the left IFG, particularly the pars opercularis, has been linked to PP (Tremblay et al., 2004; Shalom and Poeppel, 2008; Friederici, 2002; Hagoort, 2005; Costafreda et al., 2006). Our results, however, emphasize the role of the left IFG pars orbitalis in phonological fluency. Although this region has primarily been associated with SP and top-down control over conceptual representations (Badre and Wagner, 2007; Zhu et al., 2012), Hirshorn and Thompson-Schill (2006) established its involvement in auditory fMRI tasks and verbal fluency among young adults, suggesting a broader contribution to language processing. More recently, Pace et al. (2023) provided additional support for this interpretation, reporting that the left IFG pars orbitalis is significantly associated with phonological fluency performance among older adults.

In clinical contexts, PET imaging for patients with Alzheimer’s disease has explored the neural correlates of phonological fluency. Melrose et al. (2009) reported a positive correlation between hypometabolism in the IFG pars orbitalis and lower behavioral performance, a finding that aligns with ours. Similarly, structural studies in patients with PPA have shown that reduced volume in the left IFG pars orbitalis is associated with better semantic performance, suggesting a complex relationship between structure, function, and task demands (Riello et al., 2018). Furthermore, Perrone-Bertolotti et al. (2017), using fMRI and Dynamic Causal Modeling, stressed the substantial involvement of this region in both semantic and phonological tasks, with a greater relative contribution to SP. The role of the IFG pars orbitalis in PP may thus be modulated by the complexity and linguistic characteristics of the task. In our study, participants completed phonological fluency tasks in Chilean Spanish, generating words beginning with different phonemes (F, A, and S), while avoiding proper names and country names. Such constraints likely increased cognitive control demands, potentially enhancing the recruitment of the left IFG pars orbitalis (Perrone-Bertolotti et al., 2017).

In summary, our findings suggest that right-hemisphere activation among older adults may reflect a compensatory mechanism aimed at offsetting age-related declines in left frontotemporal networks (Cabeza et al., 2002). However, our data suggest that successful phonological fluency during healthy aging does not appear to require additional engagement of traditional language regions in either hemisphere. This supports the broader hypothesis that bilateral activation may represent an adaptive strategy to meet linguistic demands during older adulthood, consistent with a structural and functional reorganization of the aging brain (Neudorf et al., 2024).

## Limitations of this study

This study presents several limitations that warrant consideration. Firstly, the relatively small sample size may limit the generalizability of our findings and partly explain discrepancies with prior studies. Although all participants were right-handed, future research should incorporate left-handed individuals to examine potential differences in hemispheric lateralization patterns during language processing in aging. Secondly, syntactic processing was not directly examined, as no syntactic task or syntactic verbal fluency paradigms were included. This is relevant given previous findings stressing the role of the IFG during syntactic processing, particularly in the study by Heim et al. (2008). Including syntactic paradigms in future research may allow a more comprehensive understanding of language networks during aging. Thirdly, our study did not include in-scanner behavioral performance measures (e.g., accuracy or reaction times). Future studies incorporating these “on-task” measures could provide a more direct link between neural activation and behavioral performance during scanning, offering a clearer perspective on compensatory mechanisms. Finally, our selection of ROIs was restricted to those specified in the dual-stream model of language processing (Hickok and Poeppel, 2007), excluding occipital areas. However, recent evidence suggests that the left ventral occipitotemporal cortex, commonly referred to as the visual word form area, may contribute to language processing, particularly phonological fluency, among older adults (Jiang et al., 2017). Including such regions in future studies may provide a more complete picture of the neural architecture underlying language during aging.

## Contribution and future directions

This study advances the current understanding of the neural underpinnings of SP and PP among healthy older adults, particularly within Spanish-speaking individuals, an underrepresented population in neuroimaging research. SP and PP have been proposed as early markers for pathological aging and hold clinical relevance for differential diagnosis in dementias (Toloza-Ramírez et al., 2021; Olmos-Villaseñor et al., 2023; Julio-Ramos et al., 2024b). However, less is known about the neural organization of these processes during healthy aging.

Our findings contribute to ongoing discussions about the role of right-hemisphere regions, particularly the right IFG, in terms of supporting language processing during late-life stages. This raises important questions about whether such recruitment reflects adaptive compensation for age-related decline or the intrinsic reorganization of language networks with aging. Investigating these mechanisms for both normal and pathological aging, including conditions such as PPA, could provide valuable clinical insights.

More broadly, our results reinforce the value of fMRI for characterizing language function, especially when combined with behavioral performance and online measures. As noted by Berman et al. (2006) and Soares et al. (2016), linking functional activation with behavioral outcomes enhances the translational potential of neuroimaging for clinical applications, a perspective that aligns with the primary aims of this study.

## Conclusion

SP and PP among healthy older adults engage bilateral frontal, temporal, and parietal regions, consistent with the functional language network proposed by Hickok and Poeppel (2007). The observed associations between functional activation and behavioral performance underscore the critical contribution of right-hemisphere regions, particularly the IFG pars orbitalis, in supporting phonological tasks.

Although the IFG pars orbitalis is not traditionally implicated in PP, emerging evidence, to which our findings contribute, suggests that this region may play a previously overlooked role. Further research is needed to clarify its function in phonological fluency, especially during aging and in preclinical or clinical contexts such as mild cognitive impairment, dementia, and PPA.

Given the modest sample size, we recommend interpreting these findings with caution. Nonetheless, our study provides a foundational framework for future investigations into the neural mechanisms of language processing across the aging spectrum.

## Data Availability

The raw data supporting the conclusions of this article will be made available by the authors without undue reservation.
