# Supplementary material for: Beyond the left cerebral hemisphere: bilateral language lateralization in healthy aging and its clinical implications
Source: Front Aging Neurosci. 2025 Jul 23;17:1547162. doi: 10.3389/fnagi.2025.1547162 (PMC12325332; doi:10.3389/fnagi.2025.1547162)
Supplement: Supplementary file 1 [file Data_Sheet_1.docx]

**Supplementary Figure 1.**

**
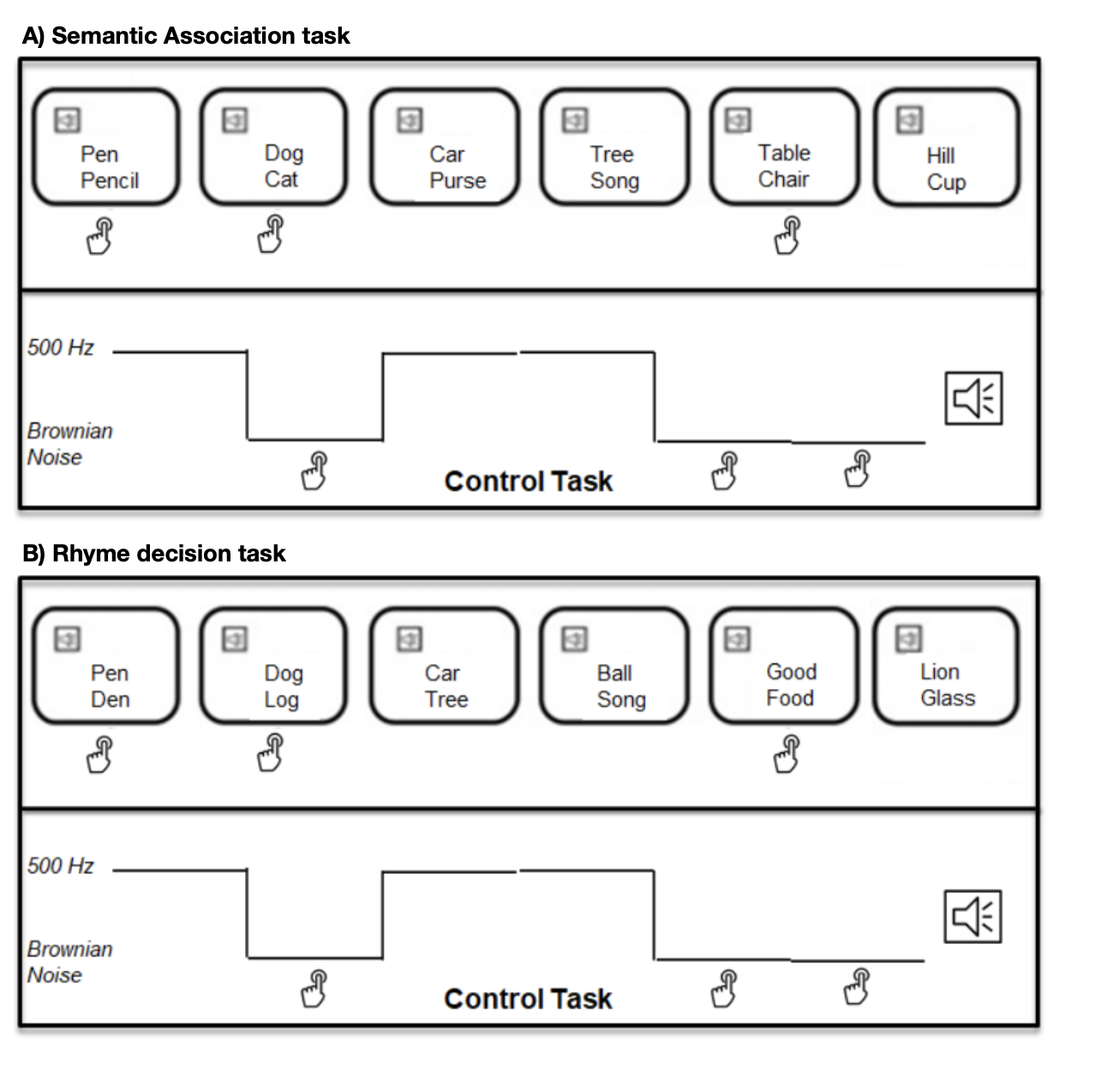
**

***Note:*** Representation of each experimental block for the semantic (top) and phonological (bottom) association tasks, each followed by a corresponding control block. The semantic block was repeated six times per participant, each time paired with its respective control task. Each experimental block lasted 21 seconds. It is important to note that the word pairs shown in the figure are representative examples; the actual stimuli used in the experiment were in Spanish. The full set of stimuli is available upon request from the corresponding author.

**Supplementary Table 4.** *Regression analysis for all significant single-predictor models.*

| Region of Interest | fMRI task | | b | df | Std Error | | *t* | *p* | R^2^ | *f*^2^ | Confidence interval (95%) | |
| --- | --- | --- | --- | --- | --- | --- | --- | --- | --- | --- | --- | --- |
|  |  |  |  |  |  |  |  |  |  |  | Lower bound | Upper bound |
|  |  |  | | | | **SCREELING SEMANTIC SCORE** | | | | | | |
| Angular gyrus – R | Semantic | | 0.20 | 24 | 0.07 | | 2.68 | **0.013*** | 0.23 | 0.30 | 0.074 | 0.374 |
| IFG pars opercularis – R | Semantic | | 0.12 | 24 | 0.05 | | 2.29 | **0.031*** | 0.18 | 0.22 | 0.050 | 0.228 |
|  |  |  | | | | **SCREELING TOTAL SCORE** | | | | | | |
| IFG pars orbitalis – R****** | Phonological | | -0.07 | 23 | 0.02 | | -2.87 | **0.008*** | 0.29 | 0.41 | -0.116 | -0.028 |
|  |  |  | | | | **PHONOLOGICAL FLUENCY: A** | | | | | | |
| IFG pars orbitalis – L | Phonological | | -0.03 | 24 | 0.01 | | -2.82 | **0.009*** | 0.25 | 0.33 | -0.056 | -0.015 |
|  |  |  | | | | **PHONOLOGICAL FLUENCY: S** | | | | | | |
| IFG pars orbitalis – L | Phonological | | -0.03 | 24 | 0.01 | | -2.09 | **0.047*** | 0.15 | 0.18 | -0.043 | -0.005 |
|  |  |  | | | | **FAS – TOTAL SCORE** | | | | | | |
| IFG pars orbitalis – L | Phonological | | -0.01 | 24 | 0.00 | | -2.39 | **0.025*** | 0.19 | 0.23 | -0.019 | -0.004 |

***Note.*** R: Right. L: Left. IFG: Inferior frontal gyrus. *Significance: p <0.05. **All reported models are single-predictor models regressing behavioral measures’ scores against ROIs activation, except for the right IFG pars orbitalis ROI (phonological task). In this case, the model controls years of schooling, therefore consisting of two predictors, with adjusted R2 reported. In this particular model, the introduced covariate was highly non-significant (*p* = 0.92).
